# Effectiveness of Mesalamine in Patients With Ulcerative Colitis: A Systematic Review

**DOI:** 10.7759/cureus.44055

**Published:** 2023-08-24

**Authors:** Yurianna Santos, Arturo P Jaramillo

**Affiliations:** 1 Sleep Medicine, The Sleep Lab of Hawaii, Kaneohe, USA; 2 General Practice, Universidad Estatal de Guayaquil, Machala, ECU

**Keywords:** fecal calprotectin, rectal bleeding, 5-asa, mesalamine-induced, ulcerative colitis (uc)

## Abstract

Ulcerative colitis (UC) management has changed significantly in the past decade. The goal is to treat the symptoms, aid tissue healing, and change the disease course to improve future outcomes. Oral or topical mesalamine (5-ASA) is a well-known UC treatment. It is the standard for starting and maintaining recovery in mild-to-moderate illnesses. The majority of patients start the treatment in the first year after diagnosis and continue it for long periods.

In this review article, PubMed/Medline, Google Scholar, and the Cochrane Library were used to search medical databases for relevant medical literature. After the articles were gathered and evaluated, 10 publications were compiled and selected using the qualifying criteria. The included articles aimed to provide an overview of 5-ASA in UC patients. According to several studies, there was no statistical relevance between various 5-ASA doses or the number of times they were taken. One study showed that 5-ASA cream preparation is better than oral preparation for patients with proctitis and proctosigmoiditis. The majority of the studies performed a follow-up to assess remission based on the use of endoscopy, fecal calprotectin, and patient symptoms during the investigations. Based on the aforementioned information, further investigation is required to ascertain the optimal approach for managing UC, with the aim of incorporating it into routine clinical procedures and enhancing our understanding of the subject matter.

## Introduction and background

Ulcerative colitis (UC) is a disease that causes inflammation of the gut mucosa. In people who have a genetic predisposition to UC, it results from a mismatch in homeostasis between the immune system of the intestines and the bacteria that reside there [1]. Individually, UC might manifest various symptoms. Stomach discomfort, rectal bleeding, less solid feces, more frequent stools, and a continual urge to go to the toilet are the most typical symptoms. Depending on the mucosal condition, this symptom might be moderate or severe [1]. People who have been diagnosed with UC are more likely to develop colon cancer [1]. Different statistical studies have shown that there are big differences in how often UC occurs. There is a wide variation in prevalence in North America, from 8.8 per million people to 23.14 per 100,000 people. Rates in Europe range anywhere from 0.97 to 57.9 per million people. In South America, the rates vary from 0.19 to 6.76 per one million individuals, whereas in Asia, the numbers range between 0.15 and 6.5 per one million. Hence, it is clear that the rate of UC is much lower in less developed countries compared to more developed countries. According to Chen et al. [1], the number of cases of UC in Asia is increasing due to the quick pace of development happening in the region. In urban areas, antibiotics are often used in both medicine and farming. The effects of urbanization on diet and how it affects gut bacteria include a drop in grain consumption (including natural fibers) and a rise in the consumption of emulsifiers and artificial sweets. Changes in eating habits such as these can cause a drop in the number of different kinds of gut microbes. People are more likely to develop UC if their gut microbiota changes as a result of air pollution, which frequently goes hand in hand with development. Researchers have found that food, social position, cleanliness habits, early-life exposure to bacteria, pollution, and other external factors can have a big effect on the human gut microbiota [1]. These factors are also very important in figuring out how well a person can handle external risks. Because of these long-term effects on gut bacteria, it has been reported that the process of development may make people more likely to develop UC [1]. Experts agree that mesalamine (5-ASA) is the best drug treatment for UC. However, side effects of ASA include sexual dysfunction, pancreatitis, cardiotoxicity, hepatotoxicity, lung manifestations, nephropathies, inflammatory reactions, and joint complaints [1]. It is very important to keep a close eye on people who take ASA for any signs of organ failure or worsening of UC [1].

When 5-ASA products were first developed, they were made to avoid the bad side effects of sulfasalazine (SSZ) use while keeping the same amount of effectiveness [2]. Proctor & Gamble, which manufactures Asacol in Cincinnati, Ohio, displays its incredible 5-ASA pills, which can dissolve at pH levels higher than 7.16, which is impressive. The same company makes Pentasa, a microsphere mixture of 5-ASA microgranules intended for release in the duodenum [2]. Lialda uses a new technology called *multimatrix* to delay and spread out the release of powerful medicines across the entire gut. Although these newer 5-ASA versions were developed to avoid the adverse effects of SSZ, they can still cause problems such as arthralgia, myalgia, flatulence, stomach pain, sickness, diarrhea, and headaches [2]. 5-ASA can also be put on the rectal mucosa area. According to Sehgal et al. [2], this might be as good as oral forms for maintaining recovery and might work faster to reduce inflammation. For treating mild-to-moderate UC, many global standards recommend taking 5-ASA by mouth or using it in combination with skin treatments [2]. Even though 5-ASA is safer than SSZ, it can cause side effects. The goal of this systematic review is to examine the good outcomes, discuss some of the most common side effects of treating UC with 5-ASA, and investigate if there is any evidence that these effects are related to the amount. The goal of this study is to give gastroenterologists advice on the different results of 5-ASA presentations and how to avoid the common side effects [2].

## Review

Review

Methodology

We performed a systematic evaluation using free full-length papers and the Preferred Reporting Items for Systematic Reviews and Meta-Analyses guidelines to describe our approach and results.

Search Strategy

The literature search was conducted on July 1st, 2023. PubMed, Google Scholar, and Cochraine Library were used to identify using the following keywords: ((“Colitis, Ulcerative/drug therapy”[Majr] OR “Colitis, Ulcerative/therapy”[Majr])) AND (“Colitis, Ulcerative/drug therapy”[Majr:NoExp] OR “Colitis, Ulcerative/therapy”[Majr:NoExp]) AND (“Mesalamine/therapeutic use”[Majr]) AND “Mesalamine/therapeutic use”[Majr:NoExp] AND ((“Aminosalicylic Acids/agonists”[Majr] OR “Aminosalicylic Acids/analysis”[Majr] OR “Aminosalicylic Acids/metabolism”[Majr] OR “Aminosalicylic Acids/pharmacology”[Majr] OR “Aminosalicylic Acids/therapeutic use”[Majr] )) AND (“Aminosalicylic Acids/agonists”[Majr:NoExp] OR “Aminosalicylic Acids/analysis”[Majr:NoExp] OR “Aminosalicylic Acids/metabolism”[Majr:NoExp] OR “Aminosalicylic Acids/pharmacology”[Majr:NoExp] OR “Aminosalicylic Acids/therapeutic use”[Majr:NoExp])

Eligibility Criteria and Study Selection

To assess eligibility, two investigators carefully read the full title and content of each paper. We selected the latest literature and articles published in the past five years, including papers written in the English language, or if the free full-text English-language translation was available. Articles were excluded if the full text of the article could not be retrieved. Articles focusing on the effectiveness of 5-ASA in patients with UC were selected. Gray literature and proposal papers were not included in this review.

Data Management

Two independent writers evaluated the articles based on titles and abstracts. Following that, significant abstracts were examined for a complete, free full-text examination. A third author evaluated the research after evaluating the chosen studies if there was any disagreement. Information from the relevant publications was then collected. The first author’s name, type, year of publication, study design, and results were gathered. Finally, duplicates were removed.

Quality Assessment

We used the Assessment of Multiple Systematic Reviews (AMSTAR) and the Cochrane Risk of Bias assessment for clinical trials and systematic reviews and meta-analyses.

Results

Search Results

**Figure 1:**
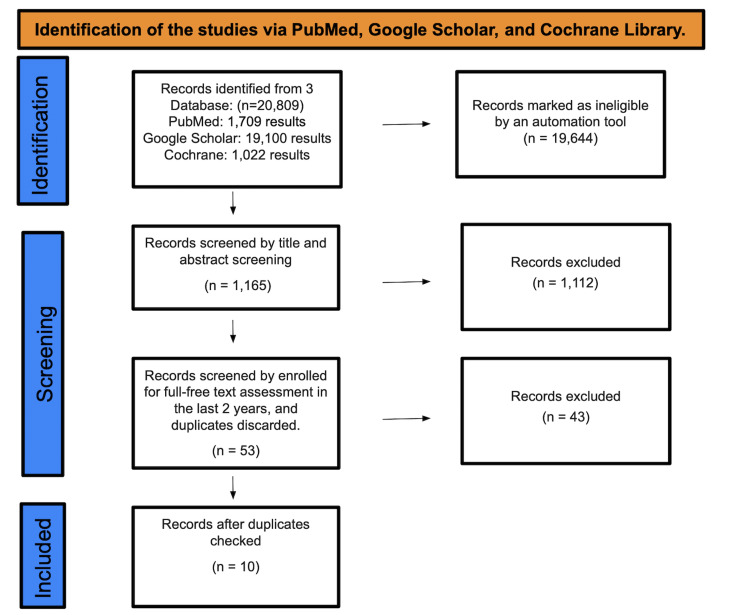
Identification of studies via databases and registers.

A total of 20,809 studies were identified after searching PubMed, Google Scholar, and the Cochrane Library. A total of 19,644 were considered ineligible by an automated tool. There were a total of 1,165 studies that underwent title and abstract screening, with 1,112 getting discarded. The remaining 53 papers were chosen by full-text evaluation in the previous five years, and after removing duplicates, resulting in the elimination of 43 studies, only 10 studies were included in this review (Figure 1).

Table 1 shows a summary and characteristics of all included studies.

**Table 1:** Characteristics of the included studies. RCT = randomized clinical trial; SRL = systematic review literature; meta-analysis = MA; UC = ulcerative colitis; 5-ASA = mesalamine; TDM = therapeutic drug monitoring; HQT = Huang Qin Tang; FC = fecal calprotectin; ARD = absolute risk difference; IBD98-M = delayed-release formulation of mesalamine; BTV = bifid triple viable

Author	Year of publication	Study design	Quality tool	Primary research	Outcome evaluation
Löwenberg et al. [3]	2023	RCT	Cochrane Risk of Bias assessment tool	In a 52-week, prospective, placebo-controlled, randomized, double-blind study, patients with active UC who were also taking 5-ASA were given TDM-guided mercaptopurine or a placebo	48.3% of mercaptopurine patients and 3/30 placebo patients met the primary aim (mean = 38.3%, 95% confidence range = 17.1–59.4, p = 0.002)
Pan et al. [4]	2022	SRL and MA	AMSTAR checklist	From inception through October 15, 2021, VIP, Embase, Wanfang Data, SinoMed, the CNKI, the Cochrane Library, and PubMed databases were thoroughly examined	HQT with 5-ASA improved UC cure rates as well as serum interleukin and immunoglobulin levels
Ma et al. [5]	2022	RCT	Cochrane Risk of Bias assessment tool	A trial of 726 UC patients treated with 5-ASA was reviewed	Improvement at week eight was linked to a 50% decrease in FC, an improvement in the global assessment by the doctor, and a decrease in rectal bleeding
Paridaens et al. [6]	2021	SRL and MA	AMSTAR checklist	From 2020 until December 2, PubMed, Embase, and Cochrane databases were searched. Sources included unpublished research	Twelve Pentasa trials included 3,674 patients. Pentasa caused and maintained clinical or endoscopic remission ARD at eight weeks better than the placebo
Barberio et al. [7]	2021	SRL and MA	AMSTAR checklist	From commencement to December 2020. Embase, Medline, and Embase Classic were used	The therapy mix of oral 5-ASA and cream 5-ASA was the best for illness recurrence. 5-ASAs were well tolerated at any dose
Tian et al. [8]	2020	SRL and MA	AMSTAR checklist	From January 2019 until October 2019, Wanfang, Embase, PubMed, the China National Knowledge Infrastructure, the Cochrane Library, and VIP searched RCTs	Fifteen studies were accepted. Thirteen studies provided clinical efficacy through endoscopy
Murray et al. [9]	2020	SRL	AMSTAR checklist	On June 11, 2019, studies were searched in three databases. Authors searched references, meeting papers, and research files for new publications	In a study that compared 5-ASA to a placebo and 5-ASA that was given once a day or as usual, the rates of serious side effects were the same for both 5-ASA and the placebo
Fiorino et al. [10]	2019	RCT	Cochrane Risk of Bias assessment tool	Randomized, phase 2a, global, parallel-group, double-blind, placebo-controlled research in moderate-to-severe UC patients	IBD98-M decreased FC and improved quality of life, but it did not statistically outperform the placebo in primary efficacy endpoints
Chen al. [1]	2019	RCT	Cochrane Risk of Bias assessment tool	From conception until October 12, 2018, the Chinese Scientific Journal Database, Cochrane Library, Chinese Knowledge, PubMed, China Biomedical Database, and Wanfang were searched	Compared to 5-ASA, the clinical effect rate, return rate, and adverse effect rate were much higher when BTV was given with 5-ASA
Sehgal et al. [2]	2018	SRL	AMSTAR checklist	The Medline database was searched for relevant works from commencement to December 1, 2017	5-ASA causes nephropathies, cardiotoxicity, respiratory problems, hepatotoxicity, inflammatory reactions, joint complaints, pancreatitis, and sexual dysfunction

Discussion

In this systematic review, we aimed to describe the effectiveness of 5-ASA from the included studies, review its side effects, and determine which approach would be useful for patients with UC. Ma et al. performed a randomized controlled study among 726 patients. By the eighth week, 436 patients had considerable ED improvement. The overall disease duration was five years, and the average initial FC level was 1,108 ug/g. The average initial Geboes score was 4.3 [11]. Despite the association between addressing ED objectives and improving long-term outcomes in UC patients, lower ED is arduous, expensive, and stressful [5]. Several observational studies have examined ED and patient outcomes. In mild-to-moderate UC, these parameters differ, emphasizing the necessity for precise, non-invasive methods to predict ED success [12,13]. Using important clinical and biological parameters before and after an eight-week 5-ASA induction treatment, a multivariable model was developed and checked internally. As previously described [5,14], the model distinguishes ED improvement from non-improvement. The study found the strongest correlation between ED improvement and a 50% drop in FC levels. The link between FC and histologic inflammation in UC is believed to be the main reason. ED responsiveness, especially after biologic treatment, is linked to FC changes [5,15].

Pentasa at 2.25-3 g/day did not differ from S-type treatment (STT) or L-type treatment (LTT) at 2.4-3 g/day in its ability to cause clinical or composite remission after eight weeks. A statistical analysis showed no significant difference between the two treatments [6]. When compared to other 5-ASAs, Pentasa did not work better at achieving clinical or composite remission after eight weeks than 5-ASA formulations of STT or LTT at 2.4-3 g/day (absolute risk difference (ARD) = 0.001, 95% CI = 0.05; p > 0.05) [6]. Pentasa and STT 5-ASA maintained remission for 12 months. Pentasa was given at a dose of 1.5-2.25 g/day, STT 5-ASA at 1.2-2.4 g/day, and SSZ at 3 g/day. ARD was 0.01 with a 95% CI of 0.07-0.08 and a p-value larger than 0.05 [6]. Barberio et al. reported that topical 5-ASA was the most effective therapy for proctitis and proctosigmoiditis in studies with a 50% participation rate. However, high-dose oral 5-ASA has continuously been in the top three, with the most trials and patients. Clinical and ED remission were better than standard and low-dose 5-ASA [7,16,17]. Oral and topical 5-ASA and high-dose oral 5-ASA were the most effective treatments for dormant UC relapses. Topical 5-ASA placed third; this has been shown to prevent recurrence in proctitis and proctosigmoiditis patients. However, small-scale studies have been done on oral-topical 5-ASA or high-dose oral 5-ASA. Unfortunately, increased oral 5-ASA dosages do not improve results [7]. 5-ASAs were well tolerated regardless of treatment strategy [7]. Tian et al. discovered that UC management must be constantly improved to provide the best therapy. As mild-to-moderate UC is on the rise globally, it is necessary [8,18]. The pathogenesis and physiology of UC are complex. UC patients have a different gut flora than healthy people [8,19]. Numerous studies found that the effects of probiotics on UC patients were unclear, but the benefits of 5-ASA were evident [8,20].

The systematic review by Murray et al. confirmed the findings of the study by Ma et al. Oral 5-ASA formulations can treat mild-to-moderately active UC. Oral 5-ASA induces remission and clinical improvement better than a placebo in active mild-to-moderate UC. Nine patients were treated to improve treatment outcomes [9]. Once-daily oral 5-ASA and traditional oral 5-ASA were equally safe. The complete data included the total number of adverse events, the number of severe adverse effects, the number of people who stopped treatment due to an adverse event, and the number of people who left the study due to an adverse event [9]. The majority of adverse events in the tests were mild-to-moderate, consistent with oral 5-ASA. UC aggravation, such as headaches, and gastrointestinal symptoms, such as flatulence, stomach pain, nausea, and diarrhea, were the most common adverse events [9]. In the study by Fiorino et al., clinical remission, the main result, was achieved by one, two, and two people in the IBD98-M 0.8 g/day, 1.2 g/day, and placebo groups, respectively, during the sixth week [10]. Responses in the treatment groups were not statistically significant compared to the placebo [10]. According to the aforementioned study, three (17.6%) patients in the IBD98-M 0.8 g/day group, five (31.3%) in the 1.2 g/day group, and three (16.7%) in the placebo group obtained clinical responses [10].

## Conclusions

We noted different outcomes from different studies. First, we concluded that 5-ASA had statistical significance in ED-confirmed clinical remission and low levels of FC. Second, the safety of the use of 5-ASA was positive, with very few cases of adverse effects. Third, there is not much change in outcomes when taking 5-ASA at different dosages or at different times daily in eight-week and 12-month studies. Finally, the administration of 5-ASA, which had excellent outcomes when used PO, was more statistically significant in the use of topical 5-ASA in patients with proctitis and proctosigmoiditis. Overall, we agree that 5-ASA has a fundamental role in the treatment of UC. Thus, more studies should be conducted to determine the best option for the use of 5-ASA in UC.

## References

[1] Chen MY, Qiu ZW, Tang HM, Zhuang KH, Cai QQ, Chen XL, Li HB (2019). Efficacy and safety of bifid triple viable plus aminosalicylic acid for the treatment of ulcerative colitis: a systematic review and meta-analysis. Medicine (Baltimore).

[2] Sehgal P, Colombel JF, Aboubakr A, Narula N (2018). Systematic review: safety of mesalazine in ulcerative colitis. Aliment Pharmacol Ther.

[3] Löwenberg M, Volkers A, van Gennep S (2023). Mercaptopurine for the treatment of ulcerative colitis: a randomized placebo-controlled trial. J Crohns Colitis.

[4] Pan C, Liu M, Li H (2022). Systematic evaluation of randomized clinical trials of Huangqin Tang in combination with mesalazine for ulcerative colitis. Evid Based Complement Alternat Med.

[5] Ma C, Jeyarajah J, Guizzetti L (2022). Modeling endoscopic improvement after induction treatment with mesalamine in patients with mild-to-moderate ulcerative colitis. Clin Gastroenterol Hepatol.

[6] Paridaens K, Fullarton JR, Travis SP (2021). Efficacy and safety of oral Pentasa (prolonged-release mesalazine) in mild-to-moderate ulcerative colitis: a systematic review and meta-analysis. Curr Med Res Opin.

[7] Barberio B, Segal JP, Quraishi MN, Black CJ, Savarino EV, Ford AC (2021). Efficacy of oral, topical, or combined oral and topical 5-aminosalicylates, in ulcerative colitis: systematic review and network meta-analysis. J Crohns Colitis.

[8] Tian C, Huang Y, Wu X, Xu C, Bu H, Wang H (2020). The efficacy and safety of mesalamine and probiotics in mild-to-moderate ulcerative colitis: a systematic review and meta-analysis. Evid Based Complement Alternat Med.

[9] Murray A, Nguyen TM, Parker CE, Feagan BG, MacDonald JK (2020). Oral 5-aminosalicylic acid for induction of remission in ulcerative colitis. Cochrane Database Syst Rev.

[10] Fiorino G, Sturniolo GC, Bossa F, Cassinotti A, di Sabatino A, Giuffrida P, Danese S (2019). A phase 2a, multicenter, randomized, double-blind, parallel-group, placebo-controlled trial of IBD98-M delayed-release capsules to induce remission in patients with active and mild to moderate ulcerative colitis. Cells.

[11] Ardizzone S, Maconi G, Russo A, Imbesi V, Colombo E, Bianchi Porro G (2006). Randomised controlled trial of azathioprine and 5-aminosalicylic acid for treatment of steroid dependent ulcerative colitis. Gut.

[12] Sood A, Midha V, Sood N, Avasthi G (2003). Azathioprine versus sulfasalazine in maintenance of remission in severe ulcerative colitis. Indian J Gastroenterol.

[13] Ma C, Sandborn WJ, D'Haens GR (2020). Discordance between patient-reported outcomes and mucosal inflammation in patients with mild to moderate ulcerative colitis. Clin Gastroenterol Hepatol.

[14] Schoepfer AM, Vavricka S, Zahnd-Straumann N, Straumann A, Beglinger C (2012). Monitoring inflammatory bowel disease activity: clinical activity is judged to be more relevant than endoscopic severity or biomarkers. J Crohns Colitis.

[15] De Vos M, Dewit O, D'Haens G (2012). Fast and sharp decrease in calprotectin predicts remission by infliximab in anti-TNF naïve patients with ulcerative colitis. J Crohns Colitis.

[16] Campieri M, Gionchetti P, Belluzzi A (1990). Topical treatment with 5-aminosalicylic in distal ulcerative colitis by using a new suppository preparation. A double-blind placebo controlled trial. Int J Colorectal Dis.

[17] Sandborn WJ, Hanauer S, Lichtenstein GR, Safdi M, Edeline M, Scott Harris M (2011). Early symptomatic response and mucosal healing with mesalazine rectal suspension therapy in active distal ulcerative colitis--additional results from two controlled studies. Aliment Pharmacol Ther.

[18] Danese S, Banerjee R, Cummings JF (2018). Consensus recommendations for patient-centered therapy in mild-to-moderate ulcerative colitis: the i Support Therapy-Access to Rapid Treatment (iSTART) approach. Intest Res.

[19] Sha S, Xu B, Wang X (2013). The biodiversity and composition of the dominant fecal microbiota in patients with inflammatory bowel disease. Diagn Microbiol Infect Dis.

[20] Derikx LA, Dieleman LA, Hoentjen F (2016). Probiotics and prebiotics in ulcerative colitis. Best Pract Res Clin Gastroenterol.

